# Synthesis, In Vitro Anti-Microbial Analysis and Molecular Docking Study of Aliphatic Hydrazide-Based Benzene Sulphonamide Derivatives as Potent Inhibitors of α-Glucosidase and Urease

**DOI:** 10.3390/molecules27207129

**Published:** 2022-10-21

**Authors:** Shoaib Khan, Shahid Iqbal, Mazloom Shah, Wajid Rehman, Rafaqat Hussain, Liaqat Rasheed, Hamad Alrbyawi, Ayed A. Dera, Mohammed Issa Alahmdi, Rami Adel Pashameah, Eman Alzahrani, Abd-ElAziem Farouk

**Affiliations:** 1 Department of Chemistry, Hazara University, Mansehra 21120, Pakistan; 2 Department of Chemistry, School of Natural Sciences (SNS), National University of Science and Technology (NUST), H-12, Islamabad 46000, Pakistan; 3 Department of Chemistry, Abbottabad University of Science and Technology (AUST), Abbottabad 22010, Pakistan; 4 Pharmaceutics and Pharmaceutical Technology Department, College of Pharmacy, Taibah University, Medina 42353, Saudi Arabia; 5 Department of Clinical Laboratory Sciences, College of Applied Medical Sciences, King Khalid University, Abha 61413, Saudi Arabia; 6 Department of Chemistry, Faculty of Science, University of Tabuk, Tabuk 71491, Saudi Arabia; 7 Department of Chemistry, Faculty of Applied Science, Umm Al-Qura University, Makkah 24230, Saudi Arabia; 8 Department of Chemistry, College of Science, Taif University, P.O. Box 11099, Taif 21944, Saudi Arabia; 9 Department of Biotechnology, College of Science, Taif University, P.O. Box 11099, Taif 21944, Saudi Arabia

**Keywords:** synthesis, sulphonamide, α-glucosidase, anti-urease, anti-microbial, SAR and molecular docking

## Abstract

A unique series of sulphonamide derivatives was attempted to be synthesized in this study using a new and effective method. All of the synthesized compounds were verified using several spectroscopic methods, including FTIR, ^1^H-NMR, ^13^C-NMR, and HREI-MS, and their binding interactions were studied using molecular docking. The enzymes urease and α-glucosidase were evaluated against each derivative (**1**–**15**). When compared to their respective standard drug such as acarbose and thiourea, almost all compounds were shown to have excellent activity. Among the screened series, analogs **5** (IC_50_ = 3.20 ± 0.40 and 2.10 ± 0.10 µM) and **6** (IC_50_ = 2.50 ± 0.40 and 5.30 ± 0.20 µM), emerged as potent molecules when compared to the standard drugs acarbose (IC_50_ = 8.24 ± 0.08 µM) and urease (IC_50_ = 7.80 ± 0.30). Moreover, an anti-microbial study also demonstrated that analogs **5** and **6** were found with minimum inhibitory concentrations (MICs) in the presence of standard drugs streptomycin and terinafine.

## 1. Introduction

Diabetes mellitus (DM) is one of the oldest known diseases worldwide. In the past, the number of patients experiencing this disease has increased every year; therefore, it is considered a lifestyle-related disease. In addition, about 366 million people have experienced this disease by 2011 reports, and the number will increase by up to 522 million by 2030 [1]. Type-II diabetes is the major type in developed countries, often caused by impaired insulin secretion as well as a reduction in the sensitivity of insulin [2,3,4,5,6]. Two enzymes are responsible for this infection: α-Amylase and α-Glucosidase. The hydrolyzing function of α-Amylase is to convert glycogen and starch to maltose and the process further carried out by α-Glucosidase which is present in the small intestine [7]. Conversions from glycogen to glucose increase the blood glucose level, hence the term post-prandial glycemia (PPHG), which is the initial indicator for type-II diabetes [8] and causes many complications in the body, such as neuropathy, nephropathy, retinopathy and cardiovascular diseases. It also damages various organs which are significant in the human body [9,10]. To control this, researchers have attempted to use different strategies to inhibit such infections, and various marketed drugs have been approved, including acarbose, voglibose and miglitol, but they have serious complications such as diarrhea, meteorism and abdominal pain [11,12].

Sulfonamide-containing drugs exhibit potent profiles due to their basic skeletons; they are also called sulpha drugs [13,14]. In a previous comparison, sulphonamide-bearing derivatives emerged as potent inhibitors with better inhibitory potential. One of the excellent inhibitors containing sulphonamide groups used for the treatment of Alzheimer’s disease, which is associated with butyrylcholinesterase (BuChE) inhibitory activity, was synthesized from previously marketed drugs containing sulphonamide derivatives and displayed excellent biological profiles at very low concentrations [15,16]. In the last few decades, sulphonamide derivatives have been extensively used as potent inhibitors against various diseases such as diabetes [17,18], psychosis [19], central nervous system (CNS) disorders [20,21], tumors [22,23,24,25,26] and different cancer treatments [27,28].Therefore, different heterocyclic moieties have been used by researchers in order to achieve fruitful results; in this regard, we synthesized and evaluated sulphonamide-containing aliphatic moieties and tested against α-Glucosidase, anti-urease, anti-bacterial and anti-fungal properties, which were found with excellent potential.

## 2. Material and Methods

### General Procedure of Aliphatic Hydrazide-Based Benzene Sulphonamide Derivatives *(**1**–**15**)*

All the required chemicals and their reagents were purchased from Sigma Aldrich (Baden-Württemberg, Germany), and the purity of the solvents were determined using distillation processes. The reaction was carried out in a fume hood, and proper investigations were maintained by checking the reaction progress after every 30 min by using thin-layer chromatography (TLC).

## 3. Results and Discussion

### Chemistry

**Scheme 1:**
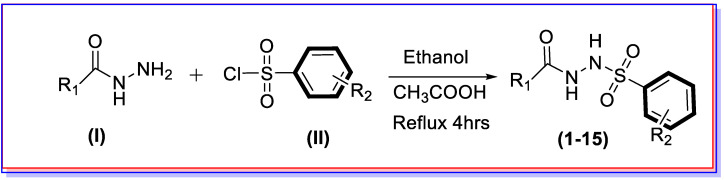
Synthesis of benzene sulphonamide derivatives (**1–15**).

The synthetic route was adopted for the synthesis of new aliphatic hydrazide-based benzene sulphonamide derivatives via a single-step reaction. Initially, a different substituted hydrazide moiety (**I**) was mixed in ethanol along with varied substituted benzene sulphonamides (**II**) followed by the addition of a few drops of acetic acid under refluxed conditions for about 4h, which afforded targeted moieties (**1–15**). All the synthesized derivatives were washed with *n*-hexane in order to remove the impurities, and fine powder was collected. Furthermore, reaction completions were confirmed by using thin-layer chromatography (TLC), and their basic skeletons were obtained through different spectroscopic techniques such as ^1^HNMR, ^13^CNMR, and HREI-MS (Scheme 1).

## 4. Spectral Analysis

The spectral analysis of the synthesized compounds, as shown (Figures S1–S6), and their interpretation values are incorporated in the Supplementary Materials.

## 5. Biological Profile

### 5.1. α-Glucosidase Inhibitory Activity

**Figure 1:**
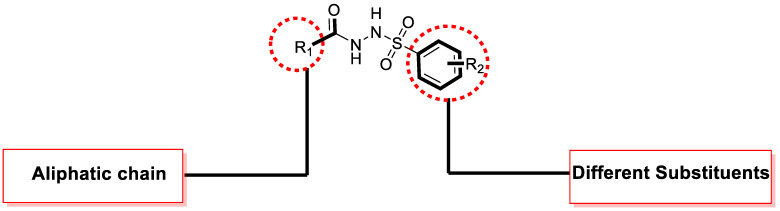
General representation of molecules.

The biological profiles exhibit the inhibitory potential of the tested scaffolds against enzymes such as α-Glucosidase, in which different substituted scaffolds showed varied ranges of inhibitory profiles. The same substituted scaffolds attached to different positions of aromatic rings (Figure 1) and produced varying degrees of inhibitions, which might be the nature of substituents. Electron-donating groups activate the ring with further interactive properties with active sites of enzymes, while deactivating groups (electron with drawing) decrease the inhibitory profile of the molecules. In the present study, better interactions were found in electron-donating groups, e.g., tri-flouro and flouro moieties showed greater interactions through strong hydrogen bonds.

The comparison criteria were set for same substituted scaffolds attached at different positions of aromatic rings in the case of methoxy substituted scaffolds **1** (IC_50_ = 8.80 ± 0.20 µM)**, 2** (IC_50_ = 9.20 ± 0.30 µM) and **3** (_IC50_ = 8.60 ± 0.20 µM). The difference in activity profile might have been due to the position of the methoxy group. *Para* and *ortho-*substituted scaffolds (**1** and **3**) had somewhat better activity than the scaffold with the *meta-*methoxy group (**2**), which reduced the chances of interactions.

Likewise, nitro substituted scaffolds **5** (IC_50_ = 3.20 ± 0.40 µM), **7** (IC_50_ = 4.50 ± 0.80 µM) and **8** (IC_50_ = 6.70 ± 0.80 µM). Among the nitro substituted scaffolds, better activity was shown by analog **5**; it might have been the presence of the nitro group at the *ortho-*position which strongly affected the enzymatic activity. When the position of the nitro group changed to *para* (**7**) and *meta* (**8**), the potentials of molecules were observed to be lower, which might have been due to the position of the substituent and the presence of the flouro group, which increased the biological activity; therefore, analog (**5**) showed a better activity profile than analog **7** and **8** and showed a few-fold better activity than the standard drug acarbose (IC_50_ = 8.24 ± 0.08).

Similarly, Chloro substituted scaffold **4** (IC_50_ = 5.70 ± 0.70 µM)**, 9** (IC_50_ = 7.40 ± 0.10 µM) and **14** (IC_50_ = 6.50 ± 0.70 µM). These scaffolds were found with minimum inhibitory concentrations (MICs). Among the chloro substituted analogs, better potential was found in the case of **4,** while analog **9** and **14** had comparable activity to the standard drug acarbose.

In the comparison criteria, the most attractive activity was shown by analog **6** (IC_50_ = 2.50 ± 0.40 µM) among the tested series. The better potential of the molecule might have been due to the presence of the tri-flouro group at the *para-*position, showing an excellent biological profile when compared with analog **15** (IC_50_ = 7.90 ± 0.80 µM)bearing the tri-flouro group at the *ortho-*position, while the methyl moiety was at the *para*-position of the ring. The change in the activity may have been due the steric hindrance of the methyl moiety; therefore, the overall effect of the molecule was found to have lower potential. Analog **6** was considered as the most potent scaffold of the tested series. The general representation of the molecule is shown in Figure 1.

The abovementioned general structure represents the different positions of molecule with varied substitutions such as the alkyl group (R = 1) and aromatic ring (R = 2). The inhibitory profile of the synthesized derivatives is based on the attached substituents on both sides of the molecule. The electron-withdrawing group (EWG) decreased the nucleophilic character of the molecule, showing a lower inhibitory profile, while the electron-donating group (EDG) increased the negative charge by donating electrons to the ring, which dominantly enhanced the biological function of the molecules. On the other hand, the alkyl group was also involved in the inhibitory profile of the molecule, which interacted through different positions of the alkyl chain. During this study, the alkyl chain with a lower number of carbons was found to have significant activity, as in the case of derivatives **5** and **6**, while the other substituted molecules with the longest alkyl chains were found to have good to moderate activity, as shown in Table 1.

### 5.2. Anti-Urease Inhibitory Activity

Similarly, the tested compound’s anti-urease profile was seen in the presence of the common standard drug thiourea (IC_50_ = 7.80 ± 0.30). The majority of the analogs had good to better activity profiles, and some had potential that was several folds greater than that of the reference drug, but in this case, analogs **5** and **6** demonstrated the most notable activity. Analog **5** (IC_50_ = 2.10 ± 0.10) bore the nitro group at the *ortho-*position and a flouro moiety at the *para-*position which created a strong ability to inhibit the enzymatic activity. Analog **5** was ranked first in the tested series because the fluoro moiety was electron withdrawing and had the ability to activate the ring system for subsequent interactions, which might boost the interaction through hydrogen bonds. Likewise, analog **6** (IC_50_ = 5.30 ± 0.20) containing a tri-flouro group at the *para*-position of the ring also showed an excellent inhibitory profile against urease enzyme and was ranked second in the series. The presence of the tri-flouro group, which similarly interacted with the active sites of enzymes by a strong hydrogen bond, may be the better interaction also measured in this analog. The fluoro group dominated in analogs **5** and **6,** and the interaction had an inhibitory effect on urease. The remaining derivatives with different substitutions on both the aliphatic and aromatic side (R1 and R2, respectively) were found to have good to moderate inhibitory profiles. The lower activity profile might have been due to the presence of the electron-withdrawing group, the bulky group as well as the largest aliphatic chain; all of these were responsible for not involving the functionality toward active sites of targeted enzymes; therefore, these moieties showed less inhibitory profiles. Moreover, the involvement of the nitro/flouro and tri-flouro group with active site of enzymes through hydrogen bonds are the key factors for the excellent activity of analogs **5** and **6**, whileothersubstituted derivatives having largest alkyl chain might be the reason for poor activity as compares to the standard drug thiourea.

### 5.3. Anti-Microbial Activity

**Figure 2:**
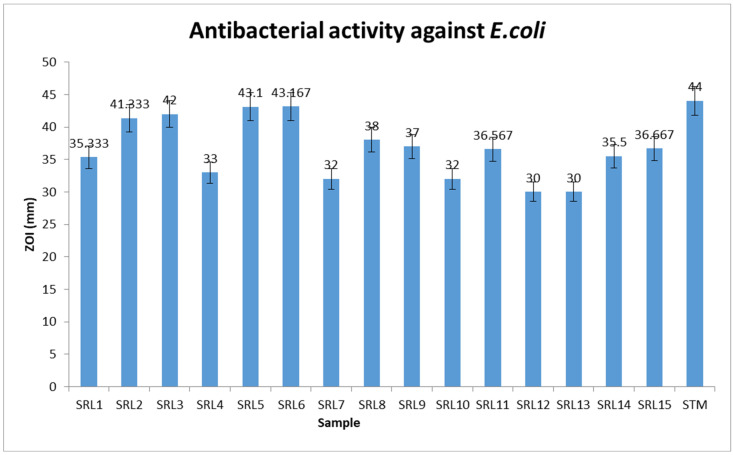
The anti-bacterial activity of scaffolds **1**–**15**.

Anti-bacterial activity has attracted substantial attention from researchers in the last few decades due to the harmful effects of bacteria [29]. The present study shows the minimum inhibitory concentration (MIC) values for the anti-microbial (anti-bacterial and anti-fungal) activity of tested compounds, which were also found to have good to moderate inhibitory profiles. Most of the scaffolds in the tested series were found to have higher inhibitory profiles in the presence of streptomycin (44% inhibition), a standard drug. The inhibitory profile of scaffolds depends on the nature and number of substituents which increase or decrease the biological potential of molecules. In addition, a few of them were found to have comparable activity with standard drugs, but in this regard, excellent activity was shown by analogs **5** and **6**. These two scaffolds reveal that the functional group located on the *para* and *ortho-*position of the aromatic ring is the critical factor for the observations of antibacterial activity. Finally, it is crucial to note that the analogs showed outstanding antibacterial selectivity against E. coli, depending on the substituents. The nature of substituents attached to aromatic rings are responsible for better or poor interaction; therefore, analogs **5** with flouro and nitro moieties and analog **6** containing a tri-flouro group were regarded as the most potent analogs in the tested series. Analog **6** showed somewhat higher inhibition (43.16%) as compared to analog **5** (43.1%);this might have been due to the presence of the tri-flouro group at the *para*-position which produced strong inhibition against *E.coli,* as shown in Figure 2.

**Figure 3:**
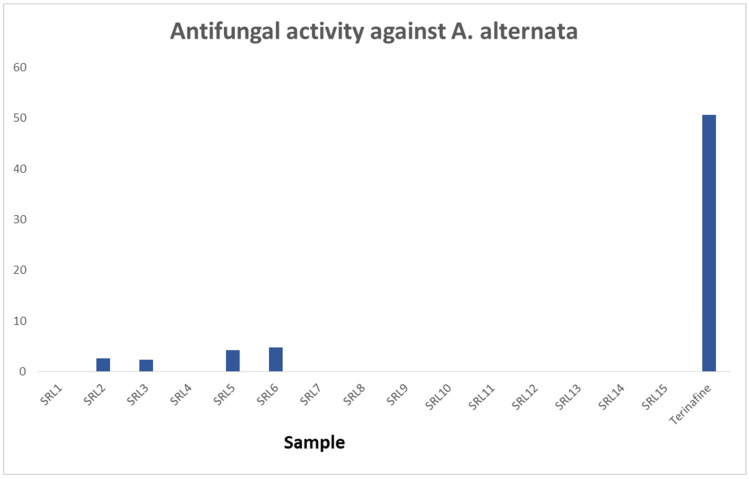
The anti-fungal activity of scaffolds **1**–**15**.

On the other hand, anti-fungal activity was observed for the synthesized compounds in the presence of the standard drug Terinafine. The majority of the compounds bearing varied substituents were found to have poor rates of inhibitions against Alternaria alternate (Fungus specie). However, a few of them were observed with somewhat comparable activity. Analogs **2**, **3**, **5** and **6** exhibited anti-fungal profiles. Here, in this study, it was found that a functional group is also necessary for a better biological profile; therefore, analogs **5** and **6** showed significant profiles compared to other substituted scaffolds, as shown in Figure 3.

### 5.4. Molecular Docking Studies

Molecular docking studies were performed in order to explore the binding interactions of ligands with active sites of enzymes. Due to better interactions, all molecules were found with somewhat comparable properties, but analogs **5** and **6** were found with strong interactions due to varied substituents (Table 2).

**Figure 4:**
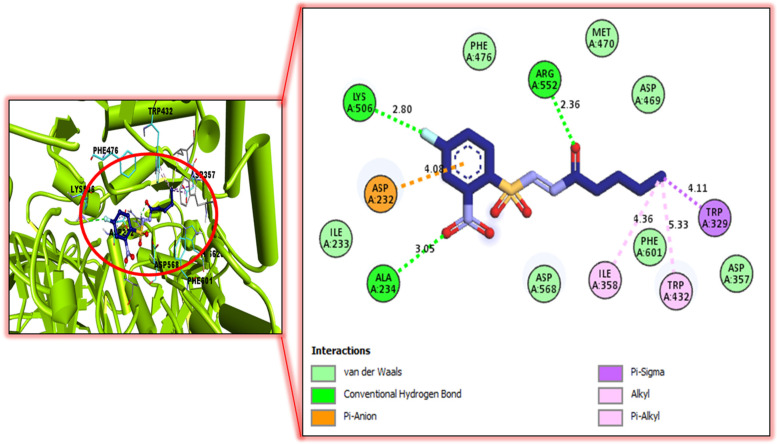
Protein–ligand interaction profile for active analog against urease represents the surface of the corresponding enzyme. The PLI profile for compound **5**.

**Figure 5:**
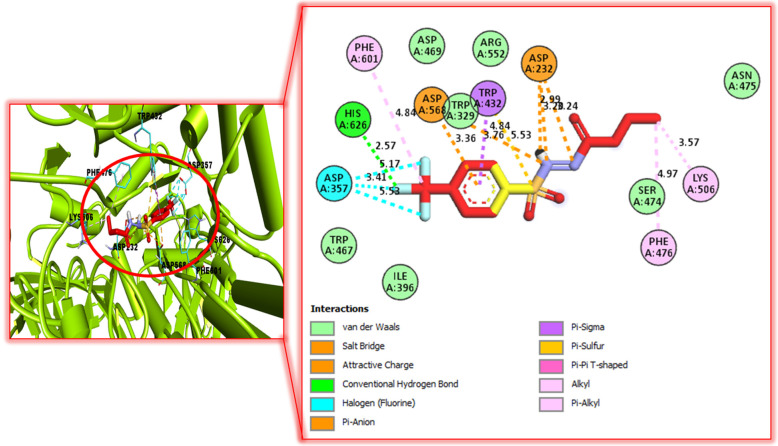
Protein–ligand interaction profiles for active analog against α-Glucosidase represent the surface of the corresponding enzyme. The PLI profile for compound **6**.

**Figure 6:**
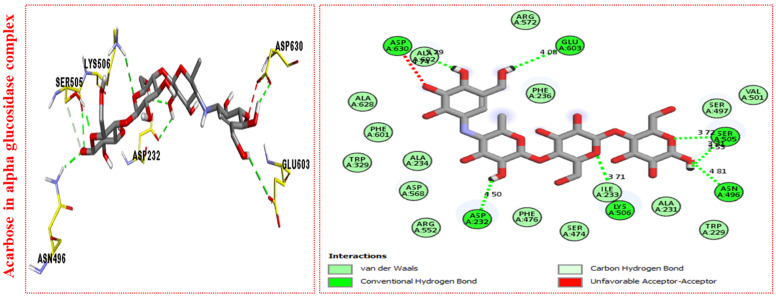
Protein–ligand interaction profiles against α-Glucosidase represent the surface of the corresponding enzyme. The PLI profile for acarbose.

In the present study, scaffolds (**5** and **6**) bearing flouro and tri-flouro moieties, respectively, were electron-withdrawing groups, but properties to make strong hydrogen bonds were found with better interactions, and their superposed surface complex structure against urease (docking score = −9.80) and α-Glucosidase enzymes (docking score = −10.70) are illustrated in Figure 4 and Figure 5, respectively. Both scaffold **5** and **6** were found to have excellent potency in vitro and better potential in silico when compared to the standard drug acarbose (docking score = −9.30), illustrated in Figure 6.

The better interactions of these ligands might have been due to the presence of attached substituents. In case of analog **5** and **6,** interactions were found such as vander Waals, conventional hydrogen bond, Pi-anion, Pi-Sigma, alkyl, Pi-alkyl, salt bridge, halogen, Pi-sulphur, etc. The further detail of docking study were summarized as in our previous articles [30,31,32,33,34,35] and the assay protocols of both the activities have been added in Supplementary file [36,37].

## 6. Conclusions

The present study was carried out for the synthesis of aliphatic hydrazide-based benzene sulphonamide derivatives. Fifteen compounds were synthesized through a single-step reaction in which most of the compounds were obtained in good yields. These scaffolds were characterized through different spectroscopic techniques such as FTIR, ^1^HNMR, ^13^CNMR and HREI-MS to confirm the basic skeletons of the synthesized compounds. Furthermore, all the synthesized scaffolds were tested against α-glucosidase and urease enzymes, and their inhibitory potentials were also checked against strains of bacteria and fungi. Alpha glucosidase and urease studies were completed in the presence of standard drugs such as acarbose (IC_50_ = 8.24 ± 0.08) and thiourea (IC_50_ = 7.80 0.30), and their biological potentials against alpha glucosidase and urease enzymes were found with good to moderate activity in this regard. For analogs **5** (IC_50_ = 3.20 ± 0.40 µM and 2.10 ± 0.10 µM, respectively) and **6** (IC_50_ = 2.50 ± 0.40 µM and 5.30 ± 0.20 µM, respectively), the anti-microbial study of these analogs were found with maximum inhibitions like **5** (43.1%) and **6** (43.16%) in the presence of streptomycin (44% inhibition) Moreover, the effective nature of these analogs was also monitored through molecular docking studies. Scaffolds **5** and **6** showed different interactions with varied ranges, and their attached substituents were found prior to all the tested analogs of the series.

## Figures and Tables

**Table 1:** Different substituted sulphonamide derivatives with inhibitory profile (**1**–**15**).

C.No	R_1_	R_2_	Alpha-Glucosidase IC_50_ (µM ± SEM)	Anti-Urease IC_50_ (µM ± SEM)
**1**	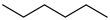	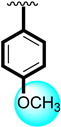	8.80 ± 0.20	17.60 ± 0.50
**2**	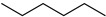	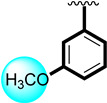	9.20 ± 0.30	21.20 ± 0.70
**3**	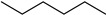	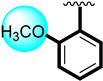	8.60 ± 0.20	12.30 ± 0.30
**4**	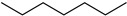	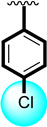	5.70 ± 0.70	8.80 ± 0.10
**5**	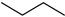	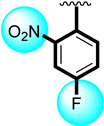	3.20 ± 0.40	2.10 ± 0.10
**6**	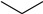	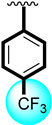	2.50 ± 0.40	5.30 ± 0.20
**7**	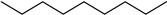	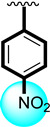	4.50 ± 0.80	9.80 ± 0.10
**8**	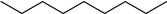	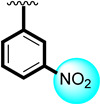	6.70 ± 0.80	13.10 ± 0.20
**9**	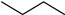	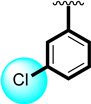	7.40 ± 0.10	11.10 ± 0.20
**10**	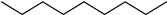	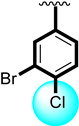	27.60 ± 0.20	23.40 ± 0.20
**11**	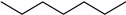	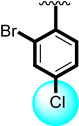	29.85 ± 0.20	25.30 ± 0.30
**12**	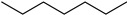	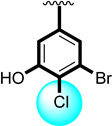	23.10 ± 0.30	16.30 ± 0.10
**13**	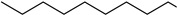	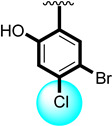	22.50 ± 0.20	18.40 ± 0.30
**14**	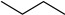	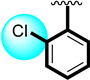	6.50 ± 0.70	7.40 ± 0.40
**15**	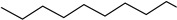	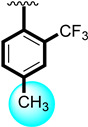	7.90 ± 0.80	8.60 ± 0.20
**Standard drug** **Acarbose and thiourea**		**IC_50_ = 8.24 ± 0.08**		**IC_50_ = 7.80 ± 0.30**

**Table 2:** The interactions of scaffolds **5** and **6** against urease and α-Glucosidase enzymes, respectively.

**Compound** **Analog-5 against** **Urease**	**Receptor**	**Interaction**	**Distance**	**Docking Score**
	LYS-A-506 H-B 2.80A ARG-A-552 H-B 2.36A TRP-A-329 Pi-Sigma 4.11A TRP-A-432 R 5.33A ILE-A-358 Pi-R 4.36A ALA-A-234 H-B 3.05A ASP-A-232 Pi-anion 4.08A			−9.80
**Analog-6 against** **α-Glucosidase**	HIS-A-626 H-B 2.57A PHE-A-601 R 2.36A ASP-A-568 Pi-anion 3.36A TRP-A-432 Pi-Sigma 4.84A ASP-A-232 Attractive charges 2.99A LYS-A-506 Pi-R 3.57A PHE-A-476 Pi-R 4.97A ASP-A-357 H-F 3.41A			−10.70
**Acarbose drug**	LYS-A-506 H-B 2.80A ASP-A-630 H-B 4.98A ALA-A-329 V.W Interaction 4.92A GLU-A-603 H-B 4.08A SER-A-505 H-B 3.72A SER-A-505 H-B 3.53A ASN-A-496 H-B 4.81A LYS-A-506 H-B 3.71A ASP-A-232 H-B 4.50A			−9.30

## Data Availability

The datasets generated during and/or analyzed during the current study are available from the corresponding author upon reasonable request.

## Supplementary Materials

The following supporting information can be downloaded at: https://www.mdpi.com/article/10.3390/molecules27207129/s1, Figures S1–S6: Spectral analysis of synthesized compounds.
