# Supplementary material for: Synthesis, In Vitro Anti-Microbial Analysis and Molecular Docking Study of Aliphatic Hydrazide-Based Benzene Sulphonamide Derivatives as Potent Inhibitors of α-Glucosidase and Urease
Source: Molecules. 2022 Oct 21;27(20):7129. doi: 10.3390/molecules27207129 (PMC9609496; doi:10.3390/molecules27207129)
Supplement: Supplementary file 1 [file molecules-27-07129-s001.zip › molecules-1948871-supplementary.pdf]

Synthesis, In Vitro Anti-Microbial Analysis and Molecular Docking Study of Aliphatic Hydrazide-Based Benzene Sulphonamide Derivatives as Potent Inhibitors of α-Glucosidase and Urease

Shoaib Khan [1,*], Shahid Iqbal [2,*], Mazloom Shah [3], Wajid Rehman [1], Rafaqat Hussain [1], Liaqat Rasheed [1], Hamad Alrbyawi [4], Ayed A. Dera [5], Mohammed Issa Alahmdi [6], Rami Adel Pashameah [7], Eman Alzahrani [8] and Abd-ElAziem Farouk [9]

[1] Department of Chemistry, Hazara University, Mansehra 21120, Pakistan
[2] Department of Chemistry, School of Natural Sciences (SNS), National University of Science and Technology (NUST), H-12, Islamabad, 46000, Pakistan
[3] Department of Chemistry, Abbottabad University of Science and Technology (AUST) Abbottabad 22010, Pakistan
[4] Pharmaceutics and Pharmaceutical Technology Department, College of Pharmacy, Taibah University, Medina 42353, Saudi Arabia
[5] Department of Clinical Laboratory Sciences, College of Applied Medical Sciences, King Khalid University, Abha 61413, Saudi Arabia
[6] Department of Chemistry, Faculty of Science, University of Tabuk, Tabuk 71491, Saudi Arabia
[7] Department of Chemistry, Faculty of Applied Science, Umm Al-Qura University, Makkah 24230, Saudi Arabia
[8] Department of Chemistry, College of Science, Taif University, P.O. Box 11099, Taif 21944, Saudi Arabia
[9] Department of Biotechnology, College of Science, Taif University, P.O. Box 11099, Taif 21944, Saudi Arabia
* Correspondence: shoaibkhanswati@gmail.com (S.K.); shahidgcs10@yahoo.com (S.I.)

2. Material and Methods

2.2. General Procedure for Molecular Docking Studies

Molecular docking study revealed the binding interaction of subjected molecules against targeted enzymes. Varied software has been used in order to explore the binding modalities of ligand with active site of enzymes. The utilized software was discovery studio visualizer (DSV) MGL tool 1.5.7 and auto Dock vina [30-35]. Using PDB code to obtain enzyme sources from www.rcsb.org protein data bank such as α-Glucosidase (**3w37**) and urease enzyme (**4ubp**). In order to investigate molecular docking study of tested scaffolds different software maintain to prepared protein as well as ligand molecule. Both the protein and ligand molecules were prepared in DSV and saved in docking folders in PDB format. In the next step protein was open in auto dock, removed water and added polar hydrogen as well as charges (kollman and gasteiger). In the last step ligand was added to prepared protein and saved both in PDBQT format in the same folder. Configuration file was set during preparation of protein along X, Y

and Z axis and saved coordinate in text format. Finally the location of the folder was identified in command prompt which generated 9-different poses for each ligand molecule. Different interactive residue has been explored might be the presence of attached substituents which either increase or decrease the interactions against targeted enzymes. Protein ligand interaction (PLI) profile has been summarized in **Table-2**.

4. Spectral Analysis

Spectra's of the some representative compounds are as given.

4.1. 3-Chloro-N'-heptanoylbenzenesulfonohydrazide

Yield 92%, m.p. 176–77ºC, Light brown. FTIR *v*, (cm^{-1}): NH (3481), Sp^{3-}CH (2916), C=O (1614), C=C (1402), S=O (1114), C-Cl (780), ^{1}HNMR (300 MHz, CDCl3): δ 9.96 (s, 1H, NH), 9.25 (s, 1H, NH), 7.98 (dd, 1H, *J* = 7.7, 1.8 Hz, Ar-H), 7.87 (s, 1H, Ar-H), 7.75 (dd, 1H, *J* = 6.8, 2.1 Hz, Ar-H), 7.39 (t, 1H, *J* = 8.0Hz, Ar-H), 2.37 (t, 2H, *J* = 7.3Hz, CH2), 1.64 (heptet, 2H, *J* = 5.7Hz, CH2), 1.41 (quintet, 6H, 3×CH2), 0.91 (t, 3H, *J* = 6.5 Hz, CH3), ^{13}C-NMR (125 MHz, DMSO-d6): δ 175.5, 140.7, 133.6, 131.9, 129.0, 125.3, 124.1, 37.3, 30.6, 28.4, 24.1, 21.3, 13.9. HR EIMS: m/z calcd for $C_{13}H_{19}ClN_2O_3S$ $[M]^+$ 318.0713; Found; 318.0641.

4.2. 4-Fluoro-N'-heptanoyl-2-nitrobenzenesulfonohydrazide

Yield 88%, m.p. 172–73ºC, Light gray. FTIR *v*, (cm^{-1}): NH (3486), Sp^{3-}CH (2927), C=O (1630), C=C (1420), N-O (1345), C-F (1250), C-N (1150), S=O (1120), C-Cl (787), ^{1}HNMR (300 MHz, CDCl3): δ 9.97 (s, 1H, NH), 9.29 (s, 1H, NH), 7.99 (d, 1H, *J* = 7.7 Hz, Ar-H), 7.89 (s, 1H, Ar-H), 7.79 (d, 1H, *J* = 6.8 Hz, Ar-H), 7.42 (t, 1H, *J* = 8.1 Hz, Ar-H), 2.39 (t, 2H, *J* = 7.1Hz, CH2), 1.65 (heptet, 2H, *J* = 5.9 Hz, CH2), 1.44 (quintet, 6H, 3×CH2), 0.94 (t, 3H, *J* = 6.1 Hz, CH3), ^{13}C-NMR (125 MHz, DMSO-d6): δ 175.7, 165.7, 147.6, 131.9, 129.2, 125.1, 124.3, 37.8, 30.0, 28.8, 24.2, 21.4, 13.7. HR EIMS: m/z calcd for $C_{13}H_{18}FN_3O_5S$ $[M]^+$ 322.2250; Found; 322.2136.

4.3. N'-Heptanoyl-4-(trifluoromethyl)benzenesulfonohydrazide

Yield 82%, m.p. 174–76ºC, Light brown. FTIR *v*, (cm^{-1}): NH (3490), Sp^{3-}CH (2950), C=O (1660), C=C (1439), C-F (1260), S=O (1129), C-Cl (770), ^{1}HNMR (300 MHz, CDCl3): δ 9.99 (s, 1H, NH), 9.32 (s, 1H, NH), 8.02 (d, 2H, *J* = 7.3 Hz, Ar-H), 7.84 (d, 2H, *J* = 7.8 Hz, Ar-H), 2.41 (t, 2H, *J* = 7.1Hz, CH2), 1.69 (heptet, 2H, *J* = 5.9 Hz, CH2), 1.48 (quintet, 6H, 3×CH2),

0.97 (t, 3H, *J* = 6.1 Hz, CH3), ^{13}C-NMR (125 MHz, DMSO-d6): δ 175.9, 139.7, 133.6, 127.4, 127.4, 126.9, 126.9, 123.6, 37.7, 30.0, 28.6, 24.1, 21.6, 13.8. HR EIMS: m/z calcd for $C_{14}H_{19}F3N_2O_3S$ $[M]^+$ 352.0917; Found; 352.0811.

4.4. 3-Chloro-N'-octanoylbenzenesulfonohydrazide

Yield 76%, m.p. 171–72°C, Light yllow. FTIR *ν*, (cm^{-1}): NH (3450), Sp3CH (2972), C=O (1616), C=C (1560), S=O (1162), C-Cl (769), ^{1}HNMR (300 MHz, CDCl3): 10.13 (s, 1H, NH), 9.42 (s, 1H, NH), 7.94 (d, 1H, *J* = 7.8Hz, Ar-H), 7.83 (s, 1H, Ar-H), 7.73 (d, 1H, *J* = 7.8Hz, Ar-H), 7.36 (t, 1H, *J* = 8.1 Hz, Ar-H), 2.34 (t, 2H, *J* = 7.5Hz, CH2), 1.68 (septet, 2H, *J* = 6.9Hz, CH2), 1.27 (quintet, J=6.3Hz, 8H, 4×CH2), 0.88 (t, 3H, *J* = 6.9 Hz, CH3), ^{13}C-NMR (125 MHz, DMSO-d6): δ 175.2, 140.5, 133.3, 131.1, 129.9, 125.4, 124.2, 37.8, 30.6, 28.3, 24.7, 21.6, 13.8, 13.4. HR EIMS: m/z calcd for $C_{14}H_{21}ClN_2O_3S$ $[M]^+$ 332.1050; Found; 332.1009.

4.5. 4-Fluoro-2-nitro-N'-pentanoylbenzenesulfonohydrazide

Yield 79%, m.p. 170–72°C, Light yellow. FTIR v, (cm-1): NH (3338), Sp3-CH (2956), C=O (1669), C=C (1599), N-O (1338), C- C-F (1210), C-N (1127), Cl (761), 1HNMR (300 MHz, CDCl3): 10.27 (s, 1H, NH), 9.52 (s, 1H, NH), 8.08 (d, 1H, J = 1.8Hz, Ar-H), 7.77 (d, J = 7.6Hz, 1H, Ar-H), 7.52 (s, 1H, Ar-H), 2.39 (t, 2H, J = 7.0Hz, CH2), 1.69 (heptet, J = 6.7Hz, 2H, CH2), 1.29 (quintet, 2H, CH2), 0.92 (t, 3H, J = 6.9 Hz, CH3), 13C-NMR (125 MHz, DMSO-d6): δ 175.3, 165.1, 147.3, 131.5, 128.1, 126.2, 123.8, 37.4, 21.6, 19.2, 13.7. HR EIMS: m/z calcd for $C_{11}H_{14}FN_3O_5S$ [M]+ 319.0589; Found; 319.0311.

4.6. N'-Butyryl-4-(trifluoromethyl)benzenesulfonohydrazide

Yield 73%, m.p. 178–79°C, Light gray. FTIR v, (cm-1): NH (3398), Sp3-CH (2964), C=O (1678), C=C (1598), C-F (1271), S=O (1173), C-Cl (733), 1HNMR (300 MHz, CDCl3): 10.08 (s, 1H, NH), 9.29 (s, 1H, NH), 8.01 (d, 2H, J = 7.9Hz, Ar-H), 7.81 (d, 2H, J = 7.5Hz, Ar-H), 2.32 (t, 2H, J = 7.4Hz, CH2), 1.64 (heptet, 2H, J = 5.7Hz, CH2), 0.91 (t, 3H, J = 6.8 Hz, CH3), 13C-NMR (125 MHz, DMSO-d6): δ 176.8, 139.5, 133.2, 127.3, 127.9, 126.7, 126.6, 122.6, 37.2, 30.6, 13.8. HR EIMS: m/z calcd for $C_{11}H_{13}F_3N_2O_3S$ [M]+ 310.0539; Found; 310.0370.

4.7. 3-Chloro-N'-decanoylbenzenesulfonohydrazide

Yield 87%, m.p. 173–74ºC, Light brown. FTIR *v*, (cm^{-1}): NH (3380), Sp3-CH (2950), C=O (1692), C=C (1581), S=O (1177), C-Cl (739), ^{1}HNMR (300 MHz, CDCl3): 10.00 (s, 1H, NH), 9.23 (s, 1H, NH), 7.96 (dd, 1H, *J* = 7.8, 1.9Hz, Ar-H), 7.83 (s, 1H, Ar-H), 7.72 (dd, 1H, *J* = 7.8, 1.7 Hz, Ar-H), 7.35 (t, 1H, *J* = 8.1Hz, Ar-H), 2.35 (t, 2H, *J* = 7.5Hz, CH2), 1.68 (heptet, 2H, *J* = 5.7Hz, CH2), 1.44 (quintet, 12H, 6×CH2), 0.89 (t, 3H, J=6.9 Hz, CH3), ^{13}C-NMR (125 MHz, DMSO-d6): δ 175.1, 140.3, 133.2, 131.0, 129.8, 125.4, 124.2, 37.8, 30.6, 28.1, 24.7, 23.4, 22.7, 21.6, 20.8, 14.4. HR EIMS: m/z calcd for $C_{16}H_{25}ClN_2O_3S$ $[M]^+$ 360.0915; Found; 360.0841.

4.8. N'-Decanoyl-4-fluoro-2-nitrobenzenesulfonohydrazide

Yield 86%, m.p. 173–75ºC, Light yellow. FTIR *v*, (cm^{-1}): NH (3394), Sp3-CH (2959), C=O (1675), C=C (1594), N-O (1360) C-F (1276), C-N (1177), S=O (1169), C-Cl (730), ^{1}HNMR (300 MHz, CDCl3): 10.06 (s, 1H, NH), 9.27 (s, 1H, NH), 7.98 (d, 1H, *J* = 7.6Hz, Ar-H), 7.84 (s, 1H, Ar-H), 7.78 (d, 1H, *J* = 7.4Hz, Ar-H), 2.38 (t, 2H, *J* = 7.4Hz, CH2), 1.69 (heptet, 2H, *J* = 5.7Hz, CH2), 1.46 (quintet, 12H, 6×CH2), 0.93 (t, 3H, *J* = 6.8 Hz, CH3), ^{13}C-NMR (125 MHz, DMSO-d6): δ 175.4, 165.6, 147.1, 131.8, 129.0, 125.7, 124.1, 37.5, 30.3, 28.9, 26.4, 26.2, 25.8, 24.1, 21.5, 12.7. HR EIMS: m/z calcd for $C_{16}H_{24}FN_3O_5S$ $[M]^+$ 389.1913; Found; 389.1341.

4.9. 4-Fluoro-2-nitro-N'-nonanoylbenzenesulfonohydrazide

Yield 77%, m.p. 171–72ºC, Light gray. FTIR *v*, (cm^{-1}): NH (3453), Sp3-CH (2992), C=O (1633), C=C (1610), N-O (1399), C-F (1270), C-N (1180), S=O (1150), C-Cl (745), ^{1}HNMR (300 MHz, CDCl3): 10.12 (s, 1H, NH), 9.42 (s, 1H, NH), 7.96 (d, 1H, *J* = 7.6Hz, Ar-H), 7.85 (s, 1H, Ar-H), 7.77 (d, 1H, *J* = 7.6Hz, Ar-H), 2.38 (t, 2H, *J* = 7.0Hz, CH2), 1.64 (septet, 2H, *J* = 8.5Hz CH2), 1.26 (quintet, 12H, 5×CH2), 0.84 (t, 3H, *J* = 6.8Hz, CH3), ^{13}C-NMR (125 MHz, DMSO-d6): δ 175.4, 165.6, 147.3, 131.8, 129.0, 125.2, 124.4, 37.6, 29.9, 28.3, 27.8, 25.1, 24.7, 22.7, 13.1. HR EIMS: m/z calcd for $C_{15}H_{22}FN_3O_5S$ $[M]^+$ 375.1293; Found; 375.0641.

4.10. 2-Chloro-N'-decanoylbenzenesulfonohydrazide

Yield 81%, m.p. 175–77ºC, Light brown. FTIR *v*, (cm^{-1}): NH (3437), Sp3-CH (2890), C=O (1629), C=C (1499), C-Cl (729), ^{1}HNMR (300 MHz, CDCl3): 9.67 (s, 1H, NH), 9.57 (s, 1H, NH), 7.84 (dd, 1H, *J* = 7.8, 1.9Hz, Ar-H), 7.8 (dd, 1H, *J* = 7.5, 1.5Hz, Ar-H), 7.46 (dt, 1H, *J* =

7.8, 1.5Hz, Ar-H), 7.42 (dt, 1H, *J* = 6.9Hz, Ar-H), 2.37 (t, 2H, *J* = 7.5Hz, CH2), 1.69 (septet, 2H, *J* = 8.7Hz CH2), 1.30 (quintet, 12H, 6×CH2), 0.89 (t, 3H, *J* = 6.9Hz, CH3), ^{13}C-NMR (125 MHz, DMSO-d6): *δ* 175.8, 140.6, 133.1, 131.3, 129.7, 125.2, 124.0, 37.6, 30.5, 28.4, 24.3, 23.4, 22.7, 21.7, 20.6, 14.3. HR EIMS: m/z calcd for $C_{16}H_{25}ClN_2O_3S$ $[M]^+$ 360.1963; Found; 360.1930.

4.11. 4-Fluoro-2-nitro-N'-octanoylbenzenesulfonohydrazide

Yield 91%, m.p. 178–79ºC, Light yllow. FTIR *v*, (cm^{-1}): NH (3391), Sp3-CH (2957), C=O (1673), C=C (1591), N-O (1344), C- C-F (1272), N (1169), S=O (1159), C-Cl (729), ^{1}HNMR (300 MHz, CDCl3): 9.69 (s, 1H, NH), 9.59 (s, 1H, NH), 7.86 (d, 1H, *J* = 7.2 Hz, Ar-H), 7.81 (d, 1H, *J* = 7.8 Hz, Ar-H), 7.47 (s, 1H, *J* = 7.1 Hz, Ar-H), 2.39 (t, 2H, *J* = 7.4Hz, CH2), 1.75 (heptet, 2H, *J* = 8.1Hz CH2), 1.37 (quintet, 12H, 4×CH2), 0.93 (t, 3H, *J* = 6.8Hz, CH3), ^{13}C-NMR (125 MHz, DMSO-d6): *δ* 175.5, 165.4, 147.3, 131.7, 129.1, 125.2, 124.8, 37.6, 30.3, 28.7, 26.5, 24.0, 21.3, 13.9. HR EIMS: m/z calcd for $C_{14}H_{20}FN_3O_5S$ $[M]^+$ 361.1920; Found; 361.1841.

4.12. N'-Octanoyl-4-(trifluoromethyl)benzenesulfonohydrazide

Yield 87%, m.p. 172–73ºC, Light brown. FTIR *v*, (cm^{-1}): NH (3405), Sp3-CH (2962), C=O (1661), C=C (1621), C-F (1240), S=O (1163), C-Cl (726), ^{1}HNMR (300 MHz, CDCl3): 9.73 (s, 1H, NH), 9.65 (s, 1H, NH), 7.89 (d, 2H, *J* = 7.5 Hz, Ar-H), 7.85 (d, 2H, *J* = 7.7 Hz, Ar-H), 2.41 (t, 2H, *J* = 7.1Hz, CH2), 1.79 (heptet, 2H, *J* = 7.1Hz CH2), 1.43 (quintet, 12H, 4×CH2), 0.99 (t, 3H, *J* = 6.4 Hz, CH3), ^{13}C-NMR (125 MHz, DMSO-d6): *δ* 175.6, 165.3, 148.5, 147.9, 131.8, 129.3, 125.4, 124.6, 37.3, 30.2, 28.6, 26.7, 24.8, 21.2, 13.5.

HR EIMS: m/z calcd for $C_{15}H_{21}F_3N_2O_3S$ $[M]^+$ 366.4132; Found; 366.4109.

4.13. 3-Chloro-N'-undecanoylbenzenesulfonohydrazide

Yield 82%, m.p. 168–69ºC, Light yellow. FTIR *v*, (cm^{-1}): NH (3330), Sp3-CH (2954), C=O (1672), C=C (1595), C-Cl (765), ^{1}HNMR (300 MHz, CDCl3): 10.23 (s, 1H, NH), 9.48 (s, 1H, NH), 8.03 (d, 1H, *J* = 1.8Hz, Ar-H), 7.72 (d, *J* = 7.8Hz, 1H, Ar-H), 7.47 (dd, 1H, *J* = 7.2, 1.2Hz, Ar-H), 7.44 (t, 1H, *J* = 7.2Hz, Ar-H), 2.34 (t, 2H, *J* = 7.5Hz, CH2), 1.66 (heptet, *J* = 6.9Hz, 2H, CH2), 1.25 (quintet, 14H, 7×CH2), 0.89 (t, 3H, *J* = 6.9 Hz, CH3), ^{13}C-NMR (125 MHz, DMSO-

d6): δ 175.7, 140.6, 133.2, 131.4, 129.6, 125.1, 124.3, 37.0, 30.4, 28.2, 26.3, 24.0, 23.5, 22.9, 21.6, 20.4, 14.2. HR EIMS: m/z calcd for $C_{17}H_{27}ClN_2O_3S$ $[M]^+$ 374.1932; Found; 374.1904.

4.14. N'-Hexanoyl-4-(trifluoromethyl)benzenesulfonohydrazide

Yield 80%, m.p. 174–76°C, Light gray. FTIR *v*, (cm^{-1}): NH (3395), Sp3-CH (2968), C=O (1637), C=C (1619), C-F (1276), S=O (1134), C-Cl (741), ^{1}HNMR (300 MHz, CDCl3): 10.14 (s, 1H, NH), 9.46 (s, 1H, NH), 7.95 (d, 2H, *J* = 7.3Hz, Ar-H), 7.89 (d, 2H, *J* = 8.3Hz, Ar-H), 2.35 (t, 2H, *J* = 7.7Hz, CH2), 1.69 (quintet, 4H, *J* = 6.0Hz, 2×CH2), 1.59 (heptet, 2H, *J* = 7.4Hz CH2), 1.30 (t, 3H, *J* = 6.1 Hz, CH3), 1.15 ^{13}C-NMR (125 MHz, DMSO-d6): δ 175.8, 139.5, 133.3, 127.2, 127.2, 126.7, 126.7, 123.9, 37.0, 30.9, 27.5, 25.2, 24.1. HR EIMS: m/z calcd for $C_{13}H_{17}F_3N_2O_3S$ $[M]^+$ 338.0417; Found; 338.0301.

4.15. 4-(Trifluoromethyl)-N'-undecanoylbenzenesulfonohydrazide

Yield 73%, m.p. 167–68°C, Light yellow. FTIR *v*, (cm^{-1}): NH (3347), Sp3-CH (2959), C=O (1672), C=C (1630), C-F (1201), C-Cl (748), ^{1}HNMR (300 MHz, CDCl3): 10.29 (s, 1H, NH), 9.54 (s, 1H, NH), 8.10 (d, 2H, *J* = 1.8Hz, Ar-H), 7.79 (d, *J* = 7.9Hz, 2H, Ar-H), 2.41 (t, 2H, *J* = 6.0Hz, CH2), 1.82 (heptet, *J* = 6.7Hz, 2H, CH2), 1.40 (quintet, 14H, 7×CH2), 1.02 (t, 3H, *J* = 6.6 Hz, CH3), ^{13}C-NMR (125 MHz, DMSO-d6): δ 175.9, 165.8, 148.4, 147.7, 131.3, 129.7, 125.2, 124.8, 37.1, 31.2, 29.6, 26.5, 25.9, 24.8, 23.4. 21.2, 20.9,13.5. HR EIMS: m/z calcd for $C_{18}H_{27}F_3N_2O_3S$ $[M]^+$ 408.1840; Found; 408.1780.

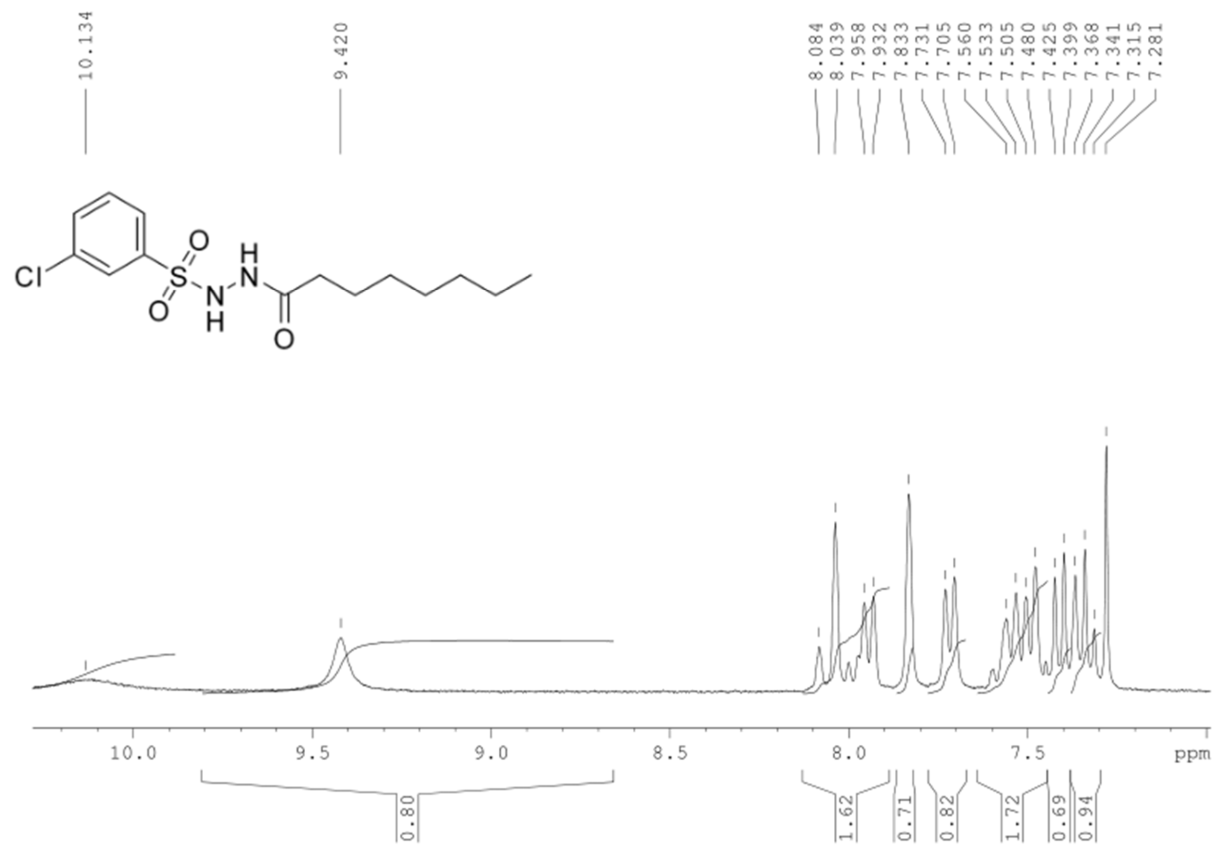

Figure S1. High resolution Proton NMR Spectrum of 3-chloro-N'-octanoylbenzenesulfonohydrazide (4.4).

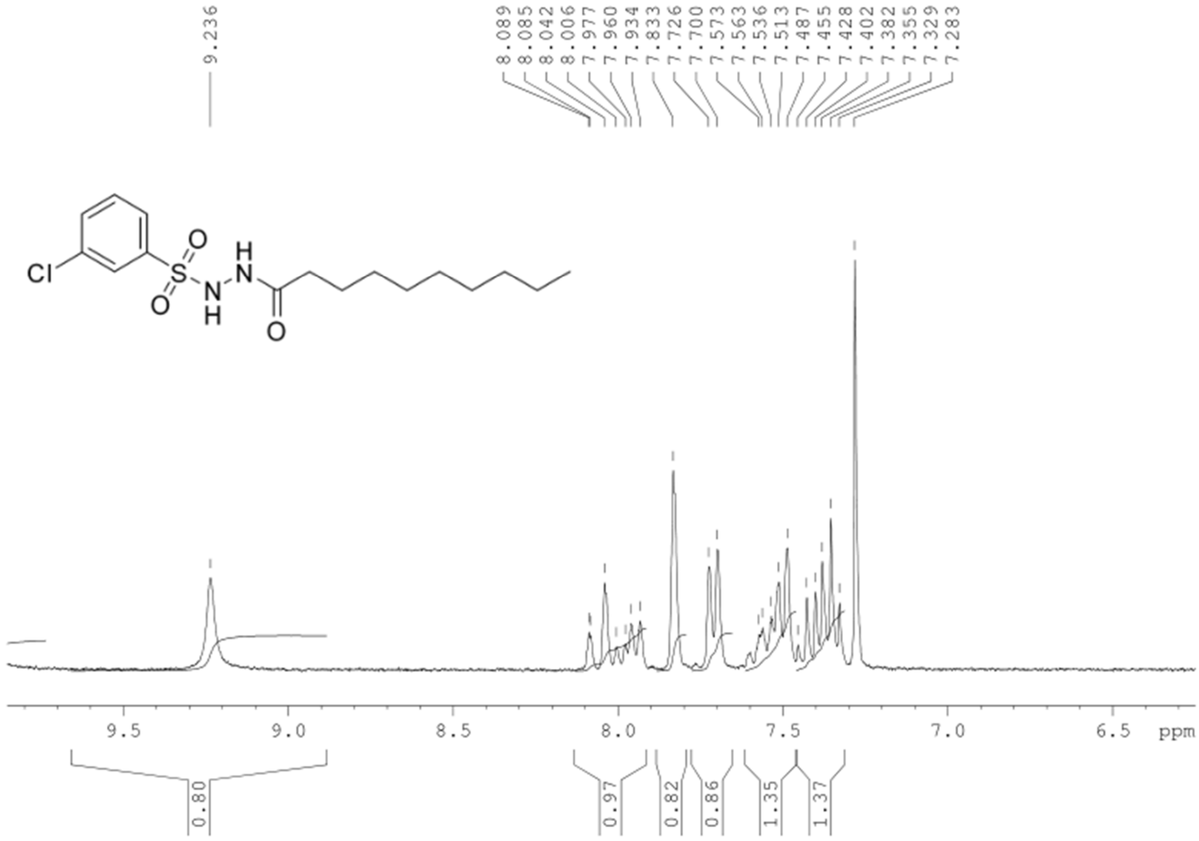

Figure S2. High resolution Proton NMR Spectrum of 3-chloro-N'-decanoylbenzenesulfonohydrazide (4.7).

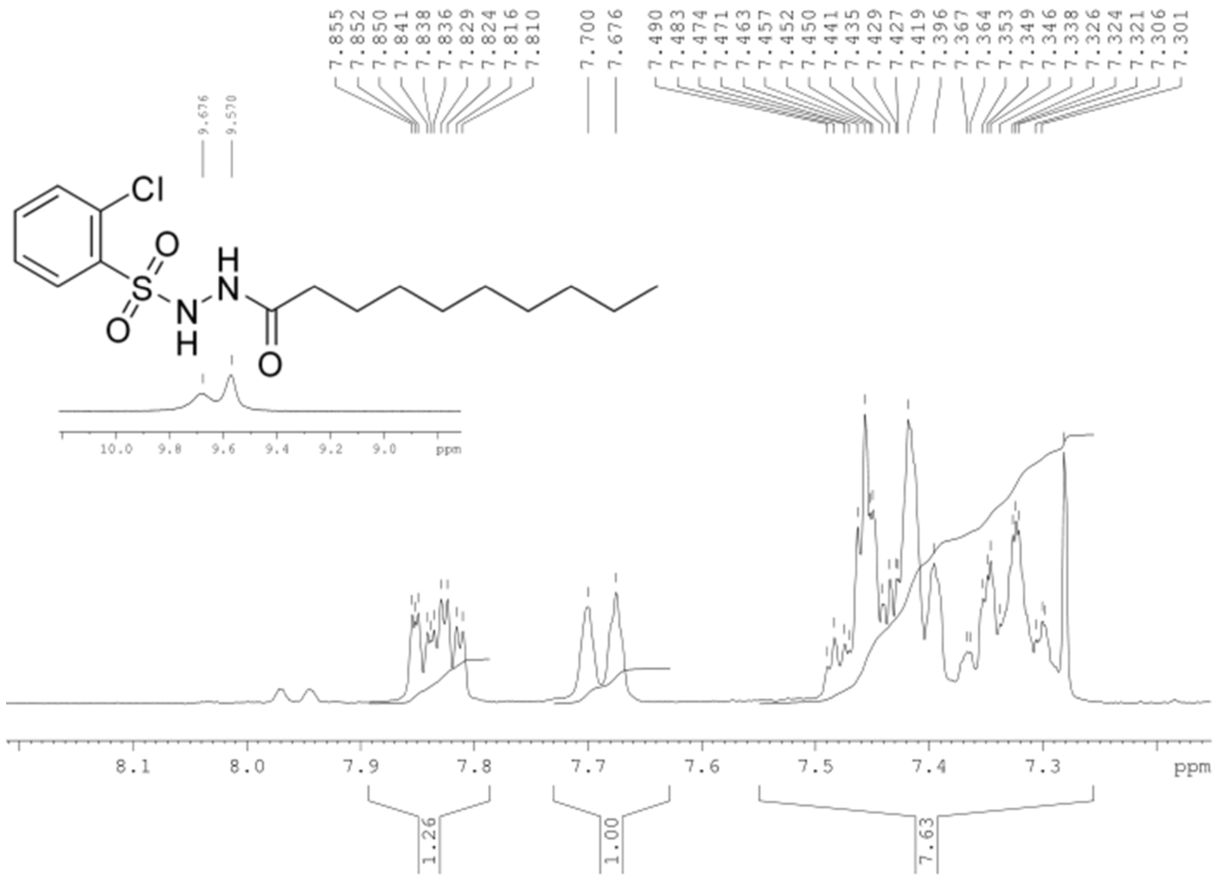

Figure S3. Low resolution Proton NMR Spectrum of 2-chloro-N'-decanoylbenzenesulfonohydrazide (4.10).

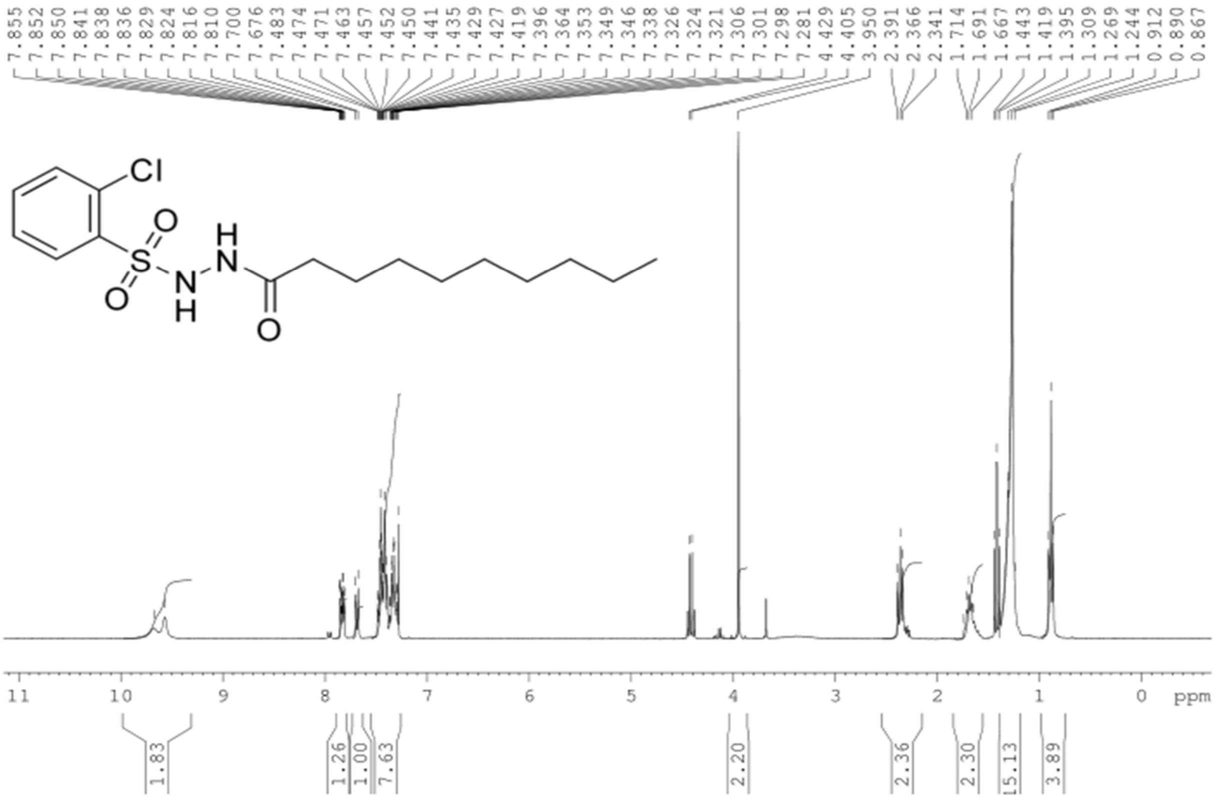

Figure S4. High resolution Proton NMR Spectrum of 2-chloro-N'-decanoylbenzenesulfonohydrazide (4.10).

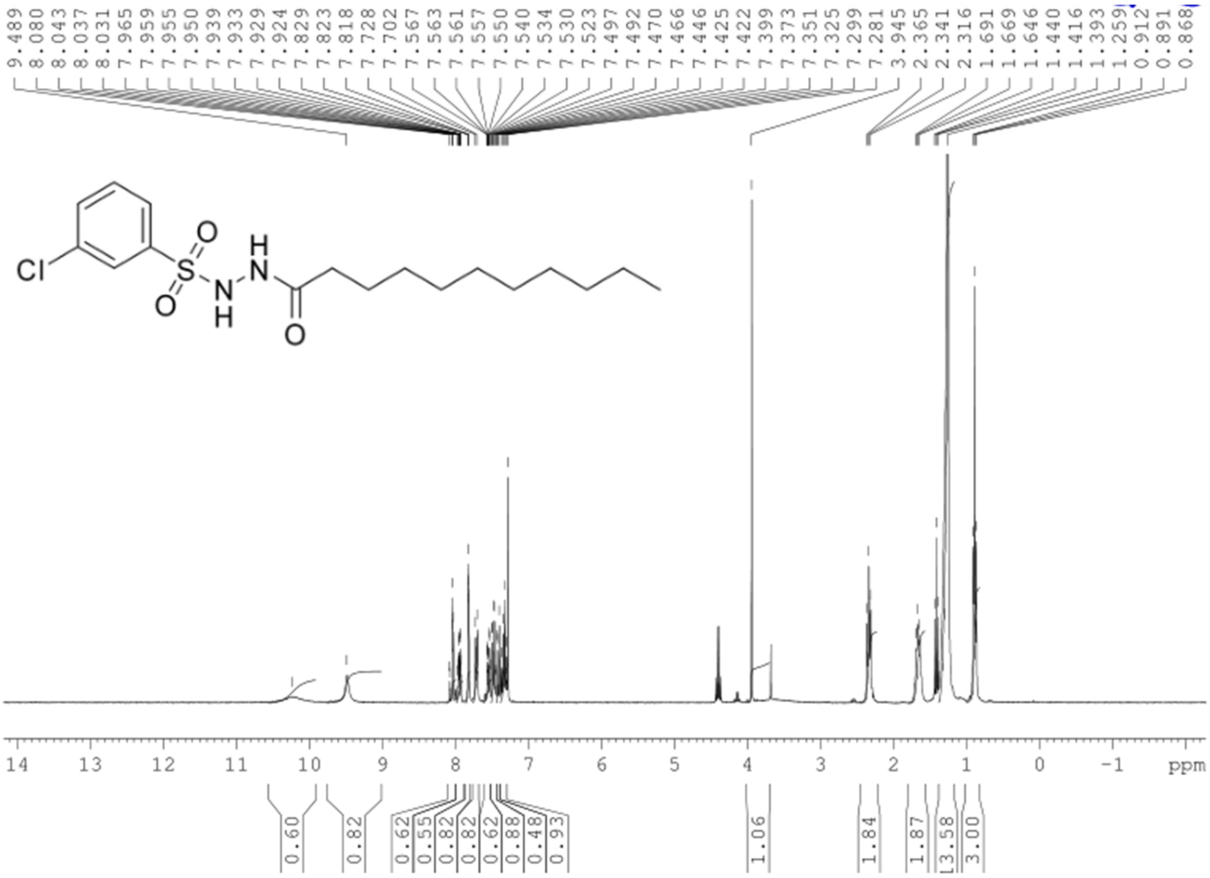

Figure S5. Low resolution Proton NMR Spectrum of 3-chloro-N'-undecanoylbenzenesulfonohydrazide (4.13).

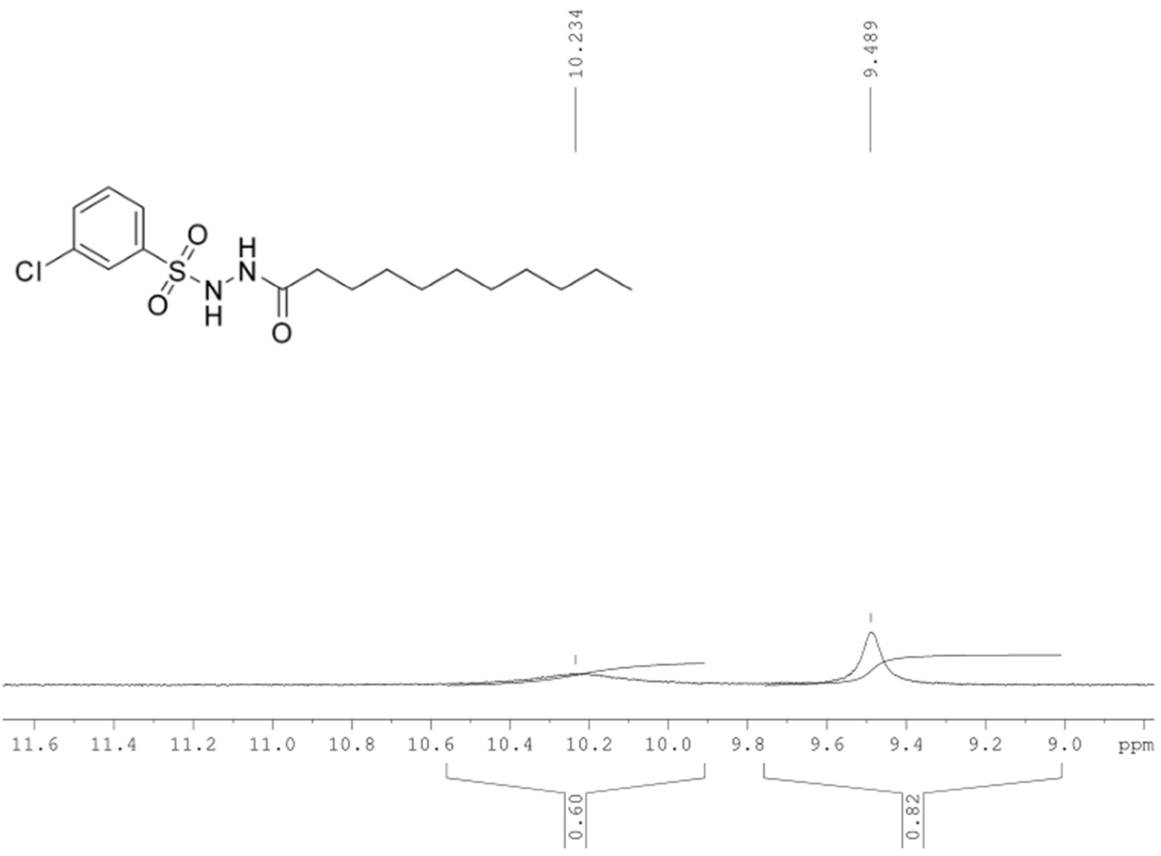

Figure S6. High resolution Proton NMR Spectrum of 3-chloro-N'-undecanoylbenzenesulfonohydrazide (4.13).

4.16. Urease Assay Protocol

The reaction mixtures, which included 25 mL of enzyme solution and 55 mL of buffers containing 100 mM urea, were incubated in 96-well plates at 30 °C for 15 min with 5 mL of the test compounds (0.5 mM concentration). The urea concentrations were adjusted from 2-24 mM for the kinetics evaluation. By quantifying ammonia production with Weatherburn's indophenol technique, urease activity was determined. [36]. To each well, 45 µL of the phenol reagent (1% w/v phenol and 0.005% w/v sodium nitroprusside) and 70 µL of the alkali reagent (0.5% w/v NaOH and 0.1% active chloride NaOCl) were added. Using a microplate reader, the rising absorbance at 630 nm was observed after 50 minutes (Molecular Device, USA). In a final volume of 200 L, each reaction was carried out three times. Using the software SoftMaxPro, the results (change in absorbance per minute) were processed (molecular Device, USA). The experiments were run at pH 6.8 throughout. The formula 100-(ODtest well/ODcontrol) 100 was

used to calculate the percentage of inhibition. Thiourea was used as the standard inhibitor for urease.

4.17. α-Glucosidase Inhibitory Assay Protocol

10 µl of the α-glucosidase enzyme, 20 µl of test samples (10-50 µg/ml) and 50 µl, of 0.1M phosphate buffer (pH=6.8), were incubated at 37°C for 15 min in a 96-well plate. 20 µl of p-nitrophenyl—D-glucopyranoside solution was further added as a substrate and again incubated for 20 min at 37 oC. Sodium carbonate (50 µl of 0.1M) was added to terminate the reaction. P-nitrophenol was liberated, during the reaction which was measured at 405nm using an ELISA microplate reader. Acarbose served as the standard. A control was prepared under similar conditions by omitting test samples. All the experiments were carried out in triplicates. The percentage inhibition was calculated by the formula. The IC50 values are tabulated in table-7 [37].

% Inhibition = [(Ac – As) /Ac] × 100 Ac-absorbance for control, As-absorbance for standard.

4.18. Molecular Docking Assay

In this study the synthesized compounds were analyzed against α-amylase and α-glucosidase enzyme. In the first step protein was prepared by using DSV by removing water molecules and already present ligand were removed save both the target protein as well as prepared ligand in PDB format. The process was further carried out in auto dock in which polar hydrogen and Kollman and Gasteiger charges were added to protein. Selected ligand was also prepared done by using torsion tree to detect root. Moreover, configuration file was generated along with X, Y and Z axis save both ligand and protein in PDBQT format in the same docking folder. At the end command prompt was used to generate varied poses of ligand thus, 9 different poses were obtained in PDBQT format. The dock protein and ligand were then open in DSV to identify the binding interaction of ligand with active sites of enzyme [33].
